# Neurophysiological Vigilance Characterisation and Assessment: Laboratory and Realistic Validations Involving Professional Air Traffic Controllers

**DOI:** 10.3390/brainsci10010048

**Published:** 2020-01-15

**Authors:** Marika Sebastiani, Gianluca Di Flumeri, Pietro Aricò, Nicolina Sciaraffa, Fabio Babiloni, Gianluca Borghini

**Affiliations:** 1 BrainSigns srl, Lungotevere Michelangelo 9, 00192 Rome, Italy; marikasebastiani@hotmail.it (M.S.); gianluca.diflumeri@uniroma1.it (G.D.F.); pietro.arico@uniroma1.it (P.A.); nicolina.sciaraffa@uniroma1.it (N.S.); fabio.babiloni@uniroma1.it (F.B.); 2 Department of Molecular Medicine, Sapienza University of Rome, Piazzale Aldo Moro 5, 00185 Rome, Italy; 3 IRCCS Fondazione Santa Lucia, Neuroelectrical Imaging and BCI Lab, Via Ardeatina 306, 00179 Rome, Italy; 4 School of Computer Science and Technology, Hangzhou Dianzi University, Hangzhou 310018, China

**Keywords:** vigilance, mental states assessment, high-resolution EEG, machine learning, psychomotor vigilance task, stepwise linear discriminant analysis, air traffic controllers, out-of-the-loop, ATM

## Abstract

Vigilance degradation usually causes significant performance decrement. It is also considered the major factor causing the out-of-the-loop phenomenon (OOTL) occurrence. OOTL is strongly related to a high level of automation in operative contexts such as the Air Traffic Management (ATM), and it could lead to a negative impact on the Air Traffic Controllers’ (ATCOs) engagement. As a consequence, being able to monitor the ATCOs’ vigilance would be very important to prevent risky situations. In this context, the present study aimed to characterise and assess the vigilance level by using electroencephalographic (EEG) measures. The first study, involving 13 participants in laboratory settings allowed to find out the neurophysiological features mostly related to vigilance decrements. Those results were also confirmed under realistic ATM settings recruiting 10 professional ATCOs. The results demonstrated that (i) there was a significant performance decrement related to vigilance reduction; (ii) there were no substantial differences between the identified neurophysiological features in controlled and ecological settings, and the EEG-channel configuration defined in laboratory was able to discriminate and classify vigilance changes in ATCOs’ vigilance with high accuracy (up to 84%); (iii) the derived two EEG-channel configuration was able to assess vigilance variations reporting only slight accuracy reduction.

## 1. Introduction

### 1.1. Vigilance Concept: General Background

The term *vigilance* is used in the scientific literature in different ways. Psychologists and cognitive neuroscientists use the term to describe the ability to sustain attention over a lengthy period under monotonous stimulus [1]. The American Psychiatric Association, in 1994, provided a similar definition of vigilance, but with specific focus on attention to potential threats or dangers: hypervigilance, for example, is considered as a common feature of various anxiety disorders, including post-traumatic stress disorder [2]. Clinical neurophysiologists sometimes use the term *vigilance level* to refer to the activity of the corticothalamic networks in the sleep–wake dimension [3]. *Arousal* refers to non-specific activation of the cerebral cortex in relation to sleep–wake states and, even if vigilance is conceptually distinct from arousal, most of the research on the vigilance studied alterations in arousal through the use of subjects who were sleep-deprived, had sleep disorders, or were taking sedative medications. Furthermore, the concept of sustained attention is often used in literature as a synonymous with vigilance. *Attention* is one of the basic human cognitive abilities that allow the discrimination of relevant parts of information and to ignore others, and it usually refers to a more focused activation of the cerebral cortex that enhances information processing [4]. According to this definition, the concept of sustained attention, i.e., that attentive component referred to as the individual’s ability to maintain the focus and remain alert over prolonged periods [5], is very close to the vigilance concept, and they are usually used as synonyms [6]. 

For the purpose of this study, *vigilance* is defined as the ability of humans to maintain their focus of attention and remain alert towards external stimuli over prolonged periods [7,8]. The timing and the magnitude of vigilance are affected by some cognitive aspects of the stimuli in addition to overt rewarding or motivational values. For example, psychological variables of external stimuli that affect vigilance include signal modality, intensity, duration, background, event rate, source complexity and declarative memory usage in the task [9], signal probability and regularity [6]. 

### 1.2. Current Key Research Points on Vigilance 

Nowadays, many occupations require high levels of vigilance, for example, security personnel [10], employees tasked with monitoring surveillance cameras or baggage screening experts [11], driving vehicles [12], diagnostic medical screening [13], real classroom settings [14], and industrial and air traffic control [15,16,17]. The need to remain alert and situation-aware, and to detect infrequent but critical signals is crucial in a lot of job occupations: A vigilance failure in any of these domains could have dramatic impacts. An extensive literature review revealed that vigilance performance is fragile on a given simulation source or task; the most common ubiquitous finding in vigilance research is that detection performance declines over time, and this decline is called *vigilance decrement* [18,19,20,21,22]. A study has shown that target detection performance decreases by 15% in 30 min during a monotonous task [23]. This reduction in cognitive efficiency will likely result in increased reaction time, error rate and may even have fatal consequences. Hence, many intervention/enhancement techniques were examined as a potential solution for attenuating vigilance decrement [8]. The literature contains a large number of means for vigilance enhancement, including mental training [24], meditation [25], sports [26], caffeine [27], video games [28], nicotine [29], diet and herbal extracts [30], chewing gum [31], odor/fragrances exposure [32], transcranial Alternating Current Stimulation (tACS) [33]. Today, in many industrial and military systems, the importance of predicting vigilance dynamics for assessing human performances is largely accepted; in fact, several studies show that accidents are often the results of vigilance failures [34,35]. The reduction of vigilance and sensitivity to important signals, for example, infrequent but critical ones, was observed in the domain in which the level of automation was very high, as in aviation [36,37], nuclear power plants [38] and the Stock Market [39].

### 1.3. Research on ATCOs and OOTL

Automation is defined as the process of entirely or partially allocating the activities constituting a task usually performed by a human, a machine, or a system [40]. Over the past 50 years, automation technology has profoundly changed modern society, humans are becoming accustomed to using and interacting with sophisticated systems designed to assist them in their activities and, in addition, more radical changes are expected in the future related to automation [2]. According to the most recent annual global statistics, provided by the International Civil Aviation Organization (ICAO) [41], the total number of passengers carried rose to 4.1 billion in 2017, 7.2% higher than the previous year, while the number of departures reached 36.7 million in 2017, a 3.1% increase compared to 2016. Furthermore, the 4.1 billion airline passengers carried in 2017 is expected to grow to about 10.0 billion by 2040, and the number of departures is projected to rise to some 90 million in 2040 [42]. So, in the aviation field, in particular, in Air Traffic Control (ATC), automation is recognised as an important topic. In fact, in future, traffic flow patterns will become more complex, and situations will be harder to identify for air traffic controllers (ATCOs). To modernise ATC systems in terms of enhanced capacity, efficiency and safety, several solutions were proposed based on higher levels of automation. A high level of automation can improve the efficiency and capacity of a system, but it is even accepted that it can have negative consequences for performance and safety, for example, unjustified and excessive trust in system ability [43] and loss of operator awareness [44]. This group of difficulties is called the out-of-the-loop phenomenon (OOTL) [45,46]. The OOTL concept describes a variety of situations where an individual is uninformed of information that is known by others [2], and it can be experienced in a lot of contexts, e.g., close relationships, social networks, government domains but, in this research, the phenomenon was considered in the context of human–machine interaction. The involvement of ATCOs in control of automatic systems, and in general, of all operators, depends on the level of automation. In a manual mode, they act on objects and perform a real control while, with a high level of automation, ATCOs are simply supervising the system and are presumably ever-alert to take over manual operations in the case of automation failures. Consequently, in the near future, the increase in levels of automation will profoundly change the ATCOs’ role, and it could lead to impair their ability to intervene in system control loops and assume manual control, when needed, in overseeing the automated system. Precisely, insufficient monitoring and checking of the automated function is one important behavioural aspect of the OOTL problem. The lack of operator involvement contributes to critical human cognitive errors, i.e., failure to detect and to understand the problem and then difficulties in finding appropriate solutions, with consequent safety incidents. These two types of problems (failure-to-detect, and failure-to-understand) have been hypothesised to occur through a change in vigilance mechanisms and changes in the information available to the ATCO. In fact, vigilance research showed that humans are poorly suited for a monitoring role [47] and reported that many incidents in the aviation field [48,49] were attributed to vigilance decrement in the human operators. Hence, to summarise, reduction in vigilance level causes degradation of the monitoring process involved in the supervisory task and decreases the performance of ATCOs in failure detection and system understanding. In addition, vigilance decrement is considered one of the major causes of the OOTL, and, with the continuous increase in automation, it is important to understand the sources of difficulties in the interaction with automation and to find solutions to compensate such difficulties. The monitoring of the current vigilance of ATCOs is an important issue to quantify the phenomenon and prevent some accidents in performing monotonous tasks. 

### 1.4. The Study Rationale: Laboratory Models and Ecological Validation

To achieve more replicable and general findings about vigilance, the most appropriate approach to study the phenomenon is the laboratory setting, by adopting ad-hoc tasks and protocols created for vigilance, with full control of the conditions of participants and environment. In this study, the psychomotor vigilance task (PVT) [50] was chosen to assess vigilance in a controlled setting. The primary outcome measures of PVT performances are lapses, defined as reaction times (RT) usually exceeding 500 ms to react [51]. Lapses constitute sensitive measures of the effects of vigilance, and impairment in executive functioning is reflected even in the count of false responses (responding when no stimulus is presented). After the experiments, participants were asked to rate their perceived attentional level using questionnaires frequently employed to measure the vigilance level. Visual analogue scale (VAS), for example, is a psychometric response scale widely used for this purpose [52]. The VAS is a measurement instrument for subjective characteristics that cannot be directly measured (e.g., vigilance during the execution of a task). In vigilance studies [53], for a given task, large differences in monitoring task performance between individuals were reported while they were not consistent for different tasks [54]. According to some authors [55], the relative performance between subjects may also vary based on the nature of the task and its demands, with the conclusion that estimation of performance and questionnaires only were not enough to achieve generalisable results about vigilance. In addition, the major drawback of subjective (i.e., questionnaires) measures is that they cannot be unobtrusively administered during task execution, but are assessed at the end of the task, which compromises the accuracy and reliability of the measurement itself [56,57]. Moreover, such methods suffer from subjective biases due to self-assessment, as well as from operator-dependent judgments. Neurophysiological measures, such as the electroencephalographic signal (EEG), allow the objective assessment of the cognitive state under which the user is performing the considered task. The effectiveness of this approach has already been explored in a variety of applications ranging from human–robot interaction to training protocols assessment, car driving, and air-traffic-control itself, even by the authors of the present work [16,37,58,59,60]. In the scientific literature, it has been widely demonstrated that the higher sensitivity of neurophysiological measurement compared to conventional techniques, such as the questionnaires, is more effective as traditional methods require a larger subject sample to highlight the same effect. [61,62].

Because of the well-established advantages and potentialities of adopting neurophysiological monitoring in the operational environment [63], the present study firstly aimed at characterising human vigilance dynamics from a neurophysiological point of view, through EEG, in laboratory settings. In fact, as previously mentioned, in this way, it is possible to induce and enhance the phenomenon-related effects by controlling all the environmental variables.

Then, a second experiment was performed in highly realistic settings to extend the validity of laboratory experiment results to real applications, where it is not possible to control external variables and the phenomenon-related effects are generally overlapped in several confounding events,. In particular, ten professional air traffic controllers (ATCOs) had to manage two similar air traffic scenarios (45-minute-long) designed by employing real air traffic data from the Airport of Munchen. While one of the two scenarios was characterised by adaptive automation, the second one was mostly fully automated, thus resulting in a 45-minute-long supervising task highly prone to vigilance decrease.

The present study aimed at identifying neurophysiological evidences linked to vigilance in controlled and in realistic environment, to define and test a vigilance index based on such evidences, and finally compare the results derived from the two contexts. Then, in both cases, we selected those features (in terms of EEG channels and brain frequency bands) able to define indexes for monitoring the ATCOs’ mental state, along the execution of Air Traffic Control activities. We investigated the possibility of defining the EEG channels’ configuration with the lowest number of electrodes able to detect vigilance changes without significant performance reduction, in terms of vigilance discrimination and classification with respect to the configuration employing a higher number of electrodes. In particular, in both studies we defined some EEG channels’ configurations in which the number of electrodes was gradually reduced (from 61 to 2 in the controlled setting and from 16 to 2 in realistic environment).

## 2. Materials and Methods

This section describes the two experimental protocols, outlines the methodology for the vigilance features selection in laboratory (i.e., Experiment 1) and realistic (i.e., Experiment 2) conditions, and defines the criteria for assess the capability and reliability of the vigilance models defined in the two contexts. 

### 2.1. Experiment 1

#### 2.1.1. Experimental Group: Students

The study involved thirteen healthy participants, all students (27 ± 3 years old), recruited on a voluntary basis. In particular, 7 males and 6 females took part at the experiments. Written informed consent was obtained from each participant on paper, after the explanation of the study. The experiments were conducted following the principles outlined in the Declaration of Helsinki of 1975, as revised in 2008. It received the favourable opinion from the Ethical Committee of the Sapienza University of Rome (CE/PROG.604). Informed consent and authorisation to use the video-graphical material (i.e., photos and videos of the experiment itself) were obtained from each subject through a written and signed form, after the explanation of the study. All the participants took part in a practice session before starting with the experiment to avoid compromised results due to learning and familiarisation processes.

#### 2.1.2. Experimental Protocol: Psychomotor Vigilance Task (PVT)

**Figure 1:**
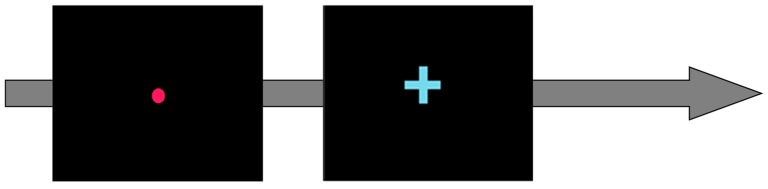
The psychomotor vigilance task (PVT) required the participants to press the space bar as fast as possible in response to a red circle which appears on the screen after a fixation cross.

The whole experimental protocol lasted about 45 min during which participants were seated at a distance of 60 cm from the monitor. The protocol consisted in three phases: in the first and third phase, participants executed the psychomotor vigilance task (namely PVT_1_ and PVT_2_) to induce vigilance degradation, while in the second phase they performed the conjunction visual search task (CNJ) to evaluate selective attention changes under three different task conditions, with increasing levels of selective attention demands. Since the aim of the present study was to characterise and identify neurophysiological features linked to vigilance changes, the CNJ dataset was not considered. Each PVT consisted of 10 min of stimuli presented on a monitor at random inter-stimulus intervals between 1–10 s. The PVT requires the participants to press the space bar on a keyboard as fast as possible in response to a red circle (target stimulus) which appears on the screen for 2 s after a fixation cross (see Figure 1). During the entire protocol, high resolution electroencephalography (HR-EEG) signals were recorded through 61 channels. In addition, the participants’ reaction time (RT) was collected in response to target stimulus to define the performance metric. Participants were asked to possibly avoid head and body movements to limit muscle artifacts. Before starting with the experiments, one minute of rest period was acquired during which the participants kept their eyes opened watching the blank monitor (OA), and then one minute keeping the eyes closed (OC) for the estimation of individual alpha frequency (IAF) [64], that is the main peak of the EEG alpha frequency band and it is usually prominent during rest condition. 

#### 2.1.3. Data Recording and Signal Processing

The HR-EEG signal was recorded by using the BrainAmp system (BrainProducts GmbH, Germany) with a sampling frequency of 250 Hz. In particular, the EEG setup included Fpz, Fp1, Fp2, AF7, AF3, AFz, AF4, AF8, F7, F5, F3, F1, Fz, F2, F4, F6, F8, FT7, FC5, FC3, FC1, FCz, FC2, FC4, FC6, FT8, T7, C5, C3, C1, Cz, C2, C4, C6, T8, TP7, CP5, CP3, CP1, CPz, CP2, CP4, CP6, TP8, P7, P5, P3, P1, Pz, P2, P4, P6, P8, PO7, PO3, POz, PO4, PO8, O1, Oz and O2, according to the 10–20 International System. All the 61 EEG channels were referred to both earlobes, grounded to both the mastoids and their impedances were kept below 10 kΩ. The acquired EEG signals were digitally band pass filtered by a $5^{th}$- order Butterworth filter (low-pass filter cut-off frequency: 40 Hz, high-pass filter cut-off frequency: 1 Hz) and by using a 50 Hz Notch filter. To remove eyeblinks and eye saccades artifacts, independent component analysis (ICA) [65] was performed. The EEG signal was segmented into epochs of 2 s, shifted of 0.125 s to obtain a high number of observations, and to respect the condition of stationarity of the EEG signal [66] to perform the spectral analysis of the signal. Specific procedures of the EEGLAB toolbox [67] were employed to identify and to remove any other kind of artifacts. In particular, the EEG epochs with a signal amplitude exceeding ±100 μV (threshold criterion) were marked as “artefact”. Then, each EEG epoch was interpolated to check the slope of the trend within the considered epoch (trend estimation). If such a slope was higher than 10 μV/s, the considered epoch was marked as “artefact”. Finally, the signal sample-to-sample difference (sample-to-sample criterion) was analysed: If such a difference was higher than 25 μV (no-physiological variation) the EEG epoch was marked as “artefact”. At the end, the EEG epochs marked as “artefact” were removed from the EEG dataset with the aim of having an artefact-free EEG dataset to perform the analyses. The power spectral density (PSD) was calculated for each EEG channel for each epoch using a Hanning window of the same length of the considered epoch (2-second long, that means 0.5 Hz of frequency resolution). Then, the EEG frequency bands of interest were defined for each subject by the estimation of the individual alpha frequency (IAF) value [64]. To have a precise estimation of the alpha peak, the participants were asked to keep their eyes closed for a minute before starting the experiment. Finally, the EEG frequency bands were defined for each participant according to the corresponding IAF, i.e., theta [IAF − 6–IAF − 2], alpha [IAF − 2–IAF + 2], beta [IAF + 2–IAF + 16] and gamma [IAF + 16–IAF + 30], and they were considered as variables for the vigilance evaluation. The PSDs corresponding to the different task conditions were normalised by subtracting the baseline condition (OA) to reduce the variability among participants.

### 2.2. Experiment 2

#### 2.2.1. Experimental Group: Professional Air Traffic Controllers

The study involved fourteen voluntary professional air traffic controllers (ATCOs), all males (45 ± 7.5 years old), from ENAV (Ente Nazionale per l’Assistenza al Volo, i.e., the Italian air navigation service provider, ANSP). The experiment took place at the Virtual Reality Lab of the University of Bologna, in Forlì (Italy) and all ATCOs were naive to the purposes of the study. The experiment was conducted following the principles outlined in the Declaration of Helsinki of 1975, as revised in 2008. It received a favourable opinion from the Ethical Committee of the University of Bologna (n. 60195). Informed consent and authorisation to use the video graphical material (i.e., photos and videos of the experiment itself) were obtained from each subject through a written and signed form, after the explanation of the study. 

#### 2.2.2. Experimental Protocol: Air Traffic Management Simulation

**Figure 2:**
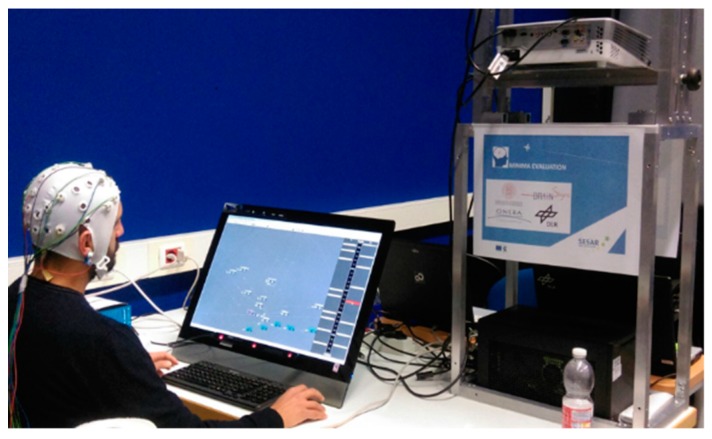
Experimental setup: Air traffic controller’s brain activity has been recorded during the execution of the air traffic management scenarios. Source: [42]. The authors own the copyright of the picture.

The air traffic controllers’ brain activity (i.e., EEG signal) was collected along the entire experimental protocol. ATCOs were asked to deal with realistic ATM scenarios (see Figure 2) using a highly automated terminal manoeuvring area (TMA). In the ATM simulator, two different levels of automation were implemented: *Level-2* was the highest level of automation, while *Level-0* corresponded to a reduced level of automation in which most of the tasks had to be manually executed. The ATCOs were instructed to monitor arriving and departing air traffic and intervene only in case of separation loss between aircraft. The real traffic data of the International Munich Airport was used and its call-signs changed between the scenarios to avoid learning effects. The data were collected during a previous study that aimed at realising an online system to prevent out-of-the-loop phenomenon [42,68] which calibrated the “vigilance and attention controller” (VAC) [69] on each ATCO before the experiment. The VAC performance has already been investigated [42] and is out of the scope of the present manuscript. Three different scenarios of 45 min were realised: a training scenario (*TRAINING*) and two experimental ones (*BASELINE* and *SOLUTION*). In the *BASELINE* scenario, the level of automation was constantly set on *Level-2* along the whole scenario to induce vigilance decreasing, caused by long monotonous tasks as the ATCOs’ role was only to monitor the air traffic [22,70]. In the *SOLUTION* scenario, the VAC adapted the level of automation on the basis of ATCOs’ vigilance level measured online via their own EEG data. Therefore, the level of automation was automatically set and eventually switched from *Level-2* to *0*, and vice versa, every 5 min based on the ATCO’s vigilance level. In addition, a scenario of 15 min, called *CALIBRATION*, was performed at the beginning of the experiments to calibrate the VAC for each ATCO. The *CALIBRATION* scenario was similar to the *BASELINE*, in which the automation level was kept on *Level-2.* Differently from the latter one, two questions about the current air traffic situation were asked during the first 5 min of the scenarios to induce high vigilance in the ATCOs. Afterward, no questions or actions were required with the aim to induce vigilance reduction in the last part of the considered scenario. The *TRAINING* scenario was introduced to allow ATCOs to familiarise themselves with the VAC and avoid results compromised by learning effects and, for such a reason, it was not considered in this study. The protocol was performed in two sessions to avoid mental fatigue due to prolonged task execution. In the first session, ATCOs executed only the *TRAINING* scenario. In the second session, the ATCOs were asked to first perform the *CALIBRATION* scenario, and successively to complete the *BASELINE* and *SOLUTION* scenarios in a random order to avoid bias in results related to the scenarios execution order. To avoid muscle artifacts, participants were asked to limit all movements, especially those of the head. In addition, before starting with the ATM tasks, ATCOs were asked to sit calmly and rest for one minute with their eyes open to acquire a baseline condition (REF) and then one minute with closed eyes (OC) for the estimation of the individual alpha frequency (IAF).

#### 2.2.3. Data Recording and Signal Processing

The EEG signal was recorded by using the g.USBamp (Guger Technologies GmbH, Austria), with a sampling frequency of 256 Hz. Sixteen traditional Ag/AgCl electrodes were placed on the prefrontal, frontal, and centro-parietal sites. In particular, the EEG setup included Fpz (it has been used only for ocular artifacts correction), AF3, AF4, AF7 AF8, Fz, F3, F4, F7, F8, CP3, CP4, Pz, P3, P4 and Oz according to the 10–20 International System. Electrodes on the earlobes were used as reference, while the ground electrode was placed on the left mastoid and the impedance of all electrodes was kept below 20 kΩ. The recorded EEG signals were digitally band-pass filtered (1–40 Hz), 5th order Butterworth filter) and the Fpz channel was used to remove eyes-blink artefacts from the signal by using the regression-based algorithm REBLINCA [71], able to work even online. After that, the signal was processed similarly to what was done in Experiment 1, therefore: (1) 2-second long epochs shifted 0.125 s [66]; (2) rejection of epochs affected by artefacts according to the three criteria described above (threshold criterion, trend estimation and Sample-to-sample criterion [67]); (3) PSD computation; (4) estimation of the IAF value [64] for defining the EEG frequency bands for each ATCO accordingly. 

### 2.3. Performed Analysis

#### 2.3.1. Vigilance Neurophysiological Characterisation

In Experiment 1, we decided to divide the PVTs into ten blocks of 1 minute to identify significant RT increasing, therefore, assuming such variable were inversely correlated with vigilance [22], the time instants of significant vigilance reduction. To identify the various parts of PVTs, a nomenclature was established as follow: PVT_1_ and PVT_2_ were, respectively, the first and the second time the participants performed the PVT (i.e., 1 or 2 subscript), while a second superscript number from 1 to 10 indicates the *n-th* 1-minute-long block of the considered PVT. For example, ${\mathrm{PVT}}_{1}^{5}$ represents the 5-th minute of the first PVT (i.e., PVT_1_). In Experiment 2, the ATM scenarios were divided into 5-minute blocks labelled with the same nomenclature of Experiment 1. For example, ${\mathrm{BAS}}^{1}\;$represents the first block (i.e., min 1–5) of *BASELINE* scenario. In both experiments, the analyses on the PSDs aimed at identifying the frequency bands and channels in which there were significant variations related to vigilance degradation. In particular, for each EEG channel and frequency bands, a non-parametric statistical test was performed on the corresponding PSDs between the high vigilance and low vigilance conditions. We excluded the central EEG channels to avoid results due to the different number of hands movements, since they are located over the motor brain cortex. In fact, the central EEG channels are widely employed for motor imagery-based brain computer interface (BCI) applications, whereby only imaging to move the left, or right, or both hands the user will be able to activate the corresponding motor brain area, that is right, left, or both central sites, respectively. In other words, the movements of the hands will lead to central brain area activations, thus to PSD differences among the central EEG channels, and the aim of our study was to characterise the Vigilance due to different cognitive demands, that is vigilance levels, and not due to different numbers of movements. The EEG channels reporting significant PSDs variation were then used to estimate the minima configuration, i.e., that EEG channels configuration constituted by only those areas revealed to be involved in the phenomenon of vigilance decreasing. In addition, the EEG channels reporting significant activity variation were compared with each other with those of the same brain area by using a non-parametric statistical test (i.e., Wilcoxon signed rank test) to avoid redundant features. The hypothesis was that if the test was statistically significant, it would mean the two considered EEG channels did not have redundant information. On the contrary, when the test was not significant, then the EEG channels would likely report redundant information. The criteria to choose which channels to keep within the minima configuration, in the latter case, was to select the EEG channel exhibiting a higher PSDs difference between the considered experimental conditions.

#### 2.3.2. Vigilance Levels Discrimination: Machine-Learning Analysis

The stepwise linear discriminant analysis (SWLDA) [71,72] regression method was chosen to identify the most significant EEG features linked to vigilance variations. The SWLDA consists of the combination of forward and backward stepwise analyses, where the input features are weighted by using ordinary least-squares regression to predict the target class labels. The method starts by creating an initial model of the discriminant function in which the most statistically significant feature is added to the model for predicting the target labels (pval*_nm_* < αENTER), where pval*_nm_* represents the *p*-value of the *m*-th feature at the *n*-th iteration. Then, at each new iteration, a new term is added to the model (if pval*_nm_* < αENTER). If there are not more features that satisfy this condition, a backward elimination analysis will be performed to remove the least statistically significant feature (if pval*_nm_* > αREMOVE) from the model. This process goes on until there are no more features satisfying the entry (αENTER) and the removal (αREMOVE) condition [73]. The employed SWLDA algorithm used the following parameters value: αENTER = 0.05 and αREMOVE = 0.1. In both experiments, for each participant, the SWLDA selected the most relevant spectral features to discriminate the vigilance levels along the experimental tasks. To estimate a global model, based on all participants, we decided to show the results in the form of features maps, i.e., each feature (i.e., PSD in specific EEG channel and brain area) was assigned a colour depending on the number of participants for which it was selected. The initial features’ domain considering all the available EEG channels, except the central and pre-frontal ones, was gradually reduced depending on the results of the statistics among the EEG channels reporting significant PSD variations between the low and high vigilance conditions to estimate minima configuration. The different vigilance models, created by providing different EEG configurations, were then validated on the testing dataset. In Experiment 1, four validations were performed: the PVT_1_ was used as training dataset and the PVT_2_ as testing dataset, and vice-versa (therefore PVT_2_ as training and PVT_1_ as testing dataset). In particular, the ${\mathrm{PVT}}_{1}^{1}$ (Class: high vigilance) and ${\mathrm{PVT}}_{1}^{9}$ (Class: low vigilance) conditions were used as training, and the ${\mathrm{PVT}}_{2}^{1}$ (Class: high vigilance) and ${\mathrm{PVT}}_{2}^{9}$ (Class: low vigilance) as testing dataset (and vice versa). Then, ${\mathrm{PVT}}_{1}^{1}$ and ${\mathrm{PVT}}_{1}^{9}$ (${\mathrm{PVT}}_{2}^{1}$ and ${\mathrm{PVT}}_{2}^{9}$) conditions were used as training and all the remaining tasks (i.e., from ${\mathrm{PVT}}_{1}^{2}$ to ${\mathrm{PVT}}_{1}^{8}$ and ${\mathrm{PVT}}_{1}^{10}$ – from ${\mathrm{PVT}}_{2}^{2}$ to ${\mathrm{PVT}}_{2}^{8}$ and ${\mathrm{PVT}}_{2}^{10}$) conditions as testing dataset. This second analysis was performed to test the method in assessing vigilance variations over time, as depicted in Section 3.4. In this regard, the first and ninth (and not the tenth and last one) blocks of both the PVT tasks have been considered as the “label conditions” of *high* (1st) and *low* (9th) vigilance classes because of the results obtained by the behavioural analysis (please refer to Section 3.1), revealing respectively the lowest and highest reaction times.

In Experiment 2, the high and low vigilance conditions of the *CALIBRATION* scenario were used as the training dataset, while the *BASELINE* and *SOLUTION* scenarios were used as the testing dataset. In this regard (please refer to Section 2.2.2 for further information), it is important to keep in mind that the CALIBRATION scenario was coherent with operational scenarios, lasting 15 min, and with the maximum level of automation (thus resulting practically in a monotonous supervising task). Therefore, in this case, the first five (1–5) and the last five (11–15) minutes were considered respectively as *high* and *low* vigilance conditions, because of scientific literature evidence [18] and of the two questions asked of the participants during the first five minutes to enhance this effect. In both the cases (laboratory and real settings), we labelled with 1 and 0 the conditions assumed as, respectively, “high vigilance” and “low vigilance” while training the SWLDA. The idea was to train the regressor with the two “extreme” conditions (therefore, labelled with 1 and 0) and to use the scores computed by the SWLDA’s discriminant function over the testing data as the vigilance index. It is not implicit, however, if the regressor was actually trained with the two range boundaries, then its score would range between 1 and 0. For the different configurations, the area under curve (AUC) and maximum accuracy (maxACC) values of the receiver operating characteristic (ROC) [74] were calculated, and finally, averaged values across the participants were estimated. To compare the discrimination (AUC) and the classification accuracy (maxACC) between all models, a non-parametric one-way ANOVA (i.e., Kruskal–Wallis’s test) was performed. The linear discriminant function (*y(t)*) was the result of the SWLDA: its output is the vigilance index (VI), and its trend over time was estimated for each participant, performing all the validations previously described for both experiments. Non-parametric statistical analysis (i.e., Kruskal–Wallis’s test) was performed to find out eventual differences in VI, and then the Tuckey’s post-hoc test was applied to estimate if there were significant difference between the considered experimental conditions of the tasks. Finally, the repeated measures correlation (rmcorr) analysis [75] was performed to estimate the correlation between the VIs and RTs in Experiment 1, to reveal the eventual relationship between the behavioural (i.e., RT) and neurophysiological (i.e., VI) measures. In Experiment 2, rmcorr was performed between the VIs of the model defined in the laboratory setting and the VIs of other models derived from the realistic environment.

## 3. Results

### 3.1. PVT: Behavioural Results

**Figure 3:**
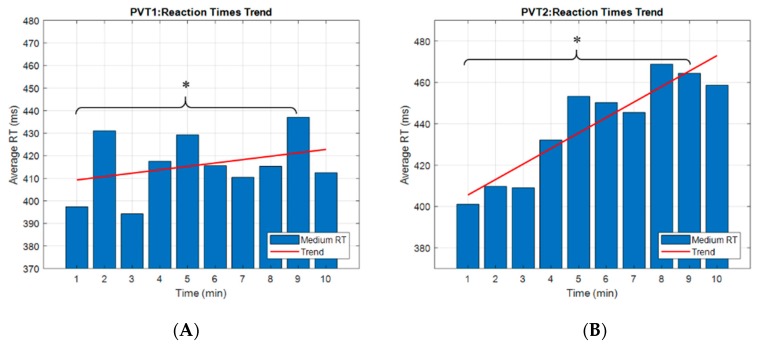
Bar graphs of the averaged reaction times (RT) across participants during the first execution of the psychomotor vigilance task (PVT_1_) (**A**) and during the second one (PVT_2_) (**B**), to evaluate the trend of the performance over time. The red line represents the trend of RT over time. In both cases, the RT trend was increased between the beginning and the end of the task. The results showed a statistical difference between the first and the ninth minute of PVT_1_ (*p* = 0.01) and PVT_2_ (*p* = 0.0017). The asterisk means that the differences were statistically significant (*p* < 0.05).

We designed our study based on the outcomes derived by Loh et al. [20], where the authors found significant vigilance degradation after 10 min of the PVT execution. However, we wanted to be sure the eventual differences coming from the neurophysiological data analyses were related to real vigilance reductions, and the only data able to support this aspect were the behavioural ones. In this regard, we first analysed the participants’ reaction time (RT) to find out if vigilance decrements occurred at the 10th minute of the PVT. The results showed an overall increase in the averaged RT in PVT_1_, as well as in PVT_2_, indicating that RT was affected by time on task (Figure 3). In particular, the statistical analysis reported a significant difference (*p* = 0.01 for PVT_1_ and *p* = 0.0017 for the PVT_2_) between the first (${\mathrm{PVT}}_{1}^{1}$ and ${\mathrm{PVT}}_{2}^{1}$) and the ninth (${\mathrm{PVT}}_{1}^{9}$ and ${\mathrm{PVT}}_{2}^{9}$) minute of the PVTs. As a consequence, such time instants were called *high* and *low* vigilance levels, respectively, and used as a training dataset for the definition of the vigilance index (VI).

### 3.2. PVT: Neurophysiological Results

**Figure 4:**
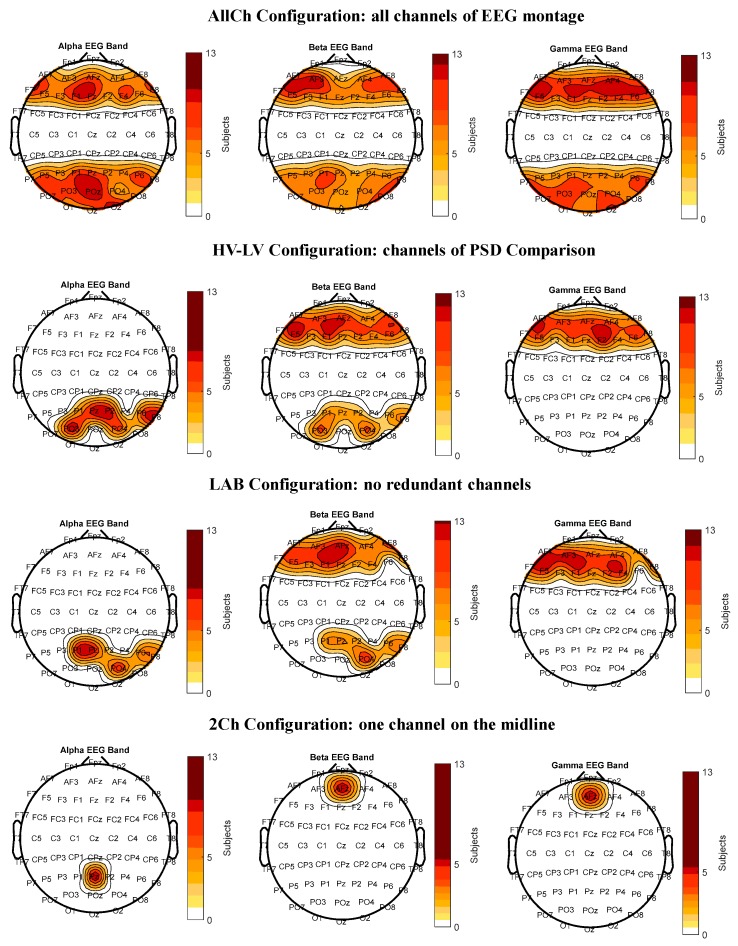
The figure reports the Features Maps of the laboratory study. The maps represent the features selected by the stepwise linear discriminant analysis (SWLDA) algorithm to characterize the vigilance using the first execution of the psychomotor vigilance task (PVT_1_) as training dataset. More intense colours indicate that the considered channel was selected for many participants.

The statistical analyses showed that the parietal alpha, frontal beta and gamma PSDs reported a significant difference between the low–high vigilance conditions. To identify the most informative EEG channels able to discriminate vigilance changes in each of these brain areas, the minima configuration was obtained with a gradual reduction of features (i.e., EEG channels) by using the stepwise linear discriminant analysis. Figure 4 shows the features included in each configuration in which EEG channels were coloured depending on the number of participants for which that feature was selected, while Table 1 reports the number of EEG channels for each configuration. The maps refer to alpha, beta and gamma bands only, in which significant results were found. The colour-code varies from white, which indicates channels were not selected for any participant, to red-colours which were associated with channels selected for an increasing number of participants. The first model is hereafter called ‘All-Channels’ (*AllCh*)*,* and it consisted of employing the SWLDA on all the 61-EEG montage channels (first from top) except the central (i.e., *FCs*, *Cs*, and *CPs*) and pre-frontal (i.e., *Fps*) channels. The ‘High Vigilance – Low vigilance’ (*HV-LV*) configuration was obtained by using the SWDLA only on the ensemble of EEG channels in which the comparison of PSD between high and low vigilance conditions reported significant changes (second image from top). Afterward, the SWLDA was used on the HV-LV configuration to investigate the possibility to further reduce the number of EEG electrodes, and thus define the ‘Laboratory’ (*LAB*) configuration (second from the bottom). Finally, the ‘2-Channels’ (*2Ch*) configuration was realised by identifying the most selected channels in each frequency band (first image from the bottom). 

### 3.3. PVT: Vigilance Discrimination and Classification Accuracy

**Figure 5:**
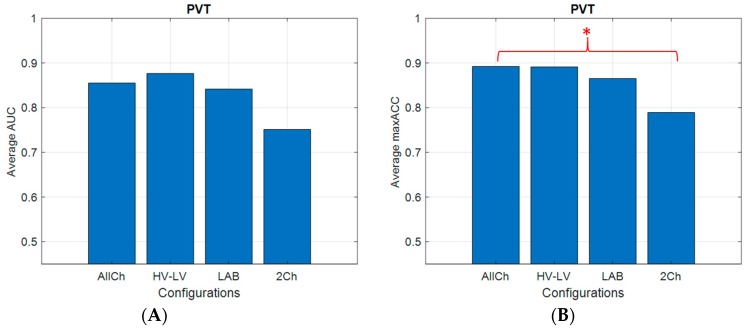
Bar plots related to the averaged area under curve (AUC) (**A**) and maximum accuracy (maxACC) (**B**) across participants for all configurations over cross-validation. The results reported that there were no significant differences across the distributions in discrimination of vigilance levels; the asterisk means that the differences were statistically significant (*p* = 0.04) between All-Channels (AllCh) and 2-Channels (2Ch) configurations in the classification of vigilance levels.

The Kruskal–Wallis’s test on the AUCs showed no statistical differences (Chi-sq (3,48) = 7.61; *p* = 0.06) among the different configurations (Figure 5). On the contrary, a significant difference was found on the maxACCs (Chi-sq (3,48) = 8.75; *p* = 0.03). In particular, Tuckey’s post-hoc tests showed that the 2Ch configuration was significantly different from the AllCh configuration (*p* = 0.04). Since the LAB configuration reported no significant differences from the AllCh configuration and its number of channels was lower than the HV-LV configuration, it was chosen for the validation in the realistic environment.

### 3.4. PVT: Vigilance Index and Correlations

**Figure 6:**
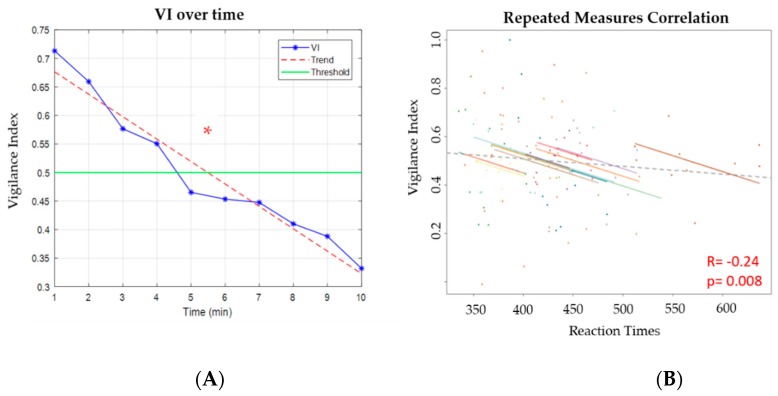
On the left, figure (**A**) reports the vigilance index (VI) trend over time for the Laboratory (LAB) configuration over cross-validation. The dotted line indicates the trend of the VI, and a threshold of 0.5 was drawn; the red asterisk indicates the statistical difference (*p* = 9 × 10^−5^) between high vigilance and low vigilance. On the right (**B**), the figure shows the significant (*p* = 0.008) correlation between the increasing Reaction Times (RT) and decreasing VI of LAB configuration.

Figure 6A reports how the VI had a decreasing trend over time, indicating an effective vigilance decrement along the PVT. Referring only to the LAB configuration, a significant difference (*p* = 9 × 10^−5^) was found between high vigilance and low vigilance levels. The rmcorr analysis confirmed the negative correlation initially assumed between the VIs and RTs and VI (R = −0.24; *p* = 0.008), as shown in Figure 6B.

### 3.5. ATM: Neurophysiological Results

**Figure 7:**
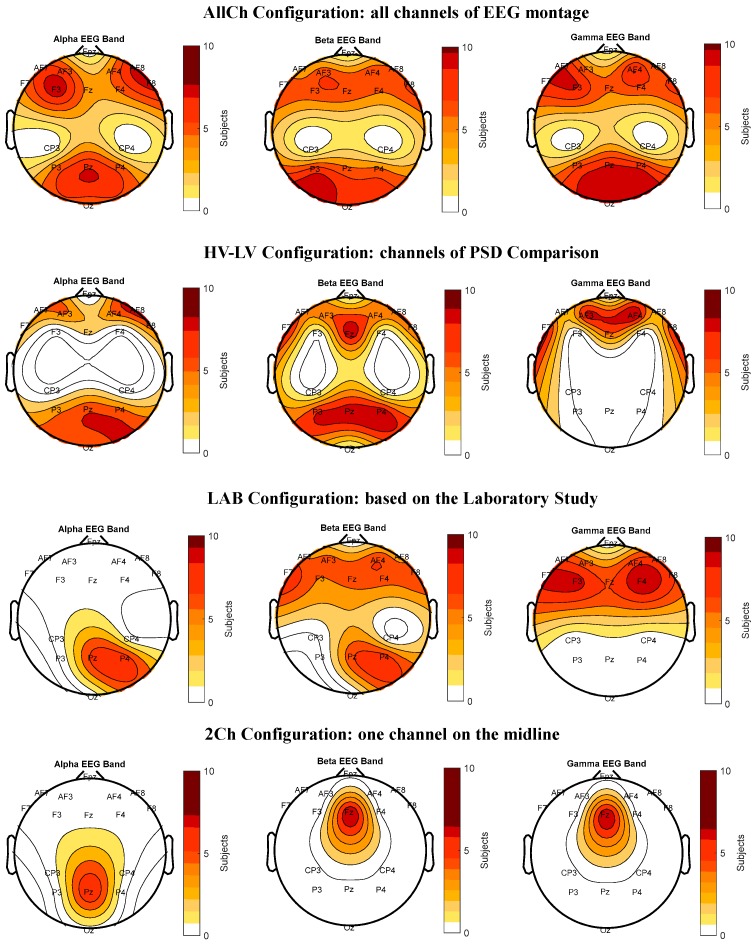
The figure reports the features maps of the realistic environment study. The maps represent the features selected by the stepwise linear discriminant analysis (SWLDA) algorithm to detect vigilance using the CALIBRATION scenario as the training dataset. More intense colours indicate that the considered channel was selected for many air traffic controllers (ATCOs).

Considering that long monotonous tasks induce vigilance decreasing [22,70], the first and the last 5-minute block of the *CALIBRATION* scenario were chosen as the training dataset for SWLDA. The parietal alpha, frontal beta and gamma bands reported significant differences between the considered blocks (i.e., high and low vigilance conditions). By applying the same procedure of Experiment 1 to reduce the features, four configurations were obtained, as shown in Figure 7, while Table 2 reports the number of EEG channels for each. In particular, the first model called hereafter AllCh included all the 16 EEG channels of the montage, except the central channels (first image from the top). The HV-LV configuration was obtained considering only the channels in which the PSDs comparison between high and low vigilance conditions reported significant changes (second image from the top). To validate the LAB configuration derived from Experiment 1 (i.e., Laboratory settings) in realistic conditions, we selected the corresponding EEG channels of the LAB configuration from those available in the 16-channel montage of Experiment 2 (second image from the bottom). Finally, the 2Ch configuration was realised by using the most selected EEG channel in each frequency band (first image from the bottom).

### 3.6. ATM: Vigilance Discrimination and Classification Accuracy

**Figure 8:**
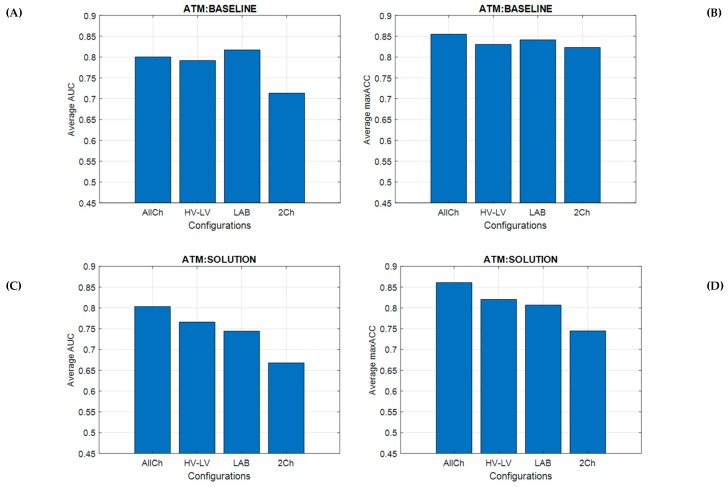
Bar plots related to the averaged area under curve (AUC) and maximum accuracy (maxACC) across air traffic controllers (ATCOs) for all configurations by using *BASELINE* (**A**,**B**) and *SOLUTION* scenarios (**C**,**D**) as the testing dataset. The results reported that there were no significant differences across the distributions in discrimination and classification of vigilance levels.

The results of Kruskal–Wallis’s tests on the AUCs and maxACCs corresponding to the different vigilance models revealed no significant differences among the EEG configurations both in the *BASELINE* (AUC: Chi-sq (3,36) = 2.74, *p* = 0.43; maxACC: Chi-sq (3,36) = 0.47, *p* = 0.93) and *SOLUTION* scenarios (AUC: Chi-sq (3,36) = 3.28, *p* = 0.35; maxACC: Chi-sq (3,36) = 5, *p* = 0.17). Averaged values across ATCOs of AUC and maxACC, for all configurations, are shown in Figure 8. 

### 3.7. ATM: Vigilance Index and Correlations

**Figure 9:**
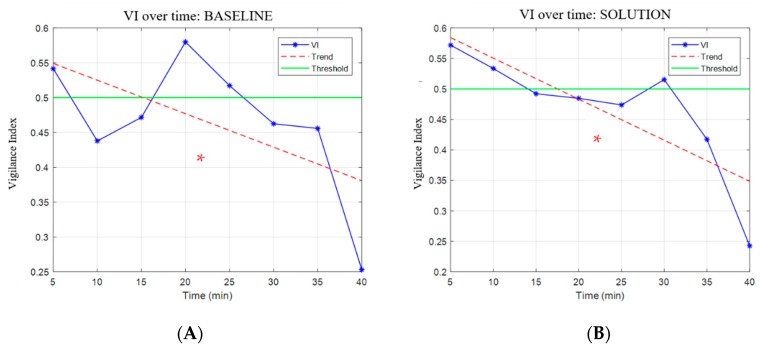
The figure reports the vigilance index (VI) trend over time for the Laboratory (LAB) configuration by using the *BASELINE* (**A**) scenario on the left and *SOLUTION* (**B**) scenario on the right. The dotted line indicates the trend of the VI, and a threshold of 0.5 is drawn. The red asterisk indicates the statistical difference (*p* = 0.004 in *BASELINE* and *p* = 8 × 10^−5^ in *SOLUTION*) between high vigilance and low vigilance.

**Figure 10:**
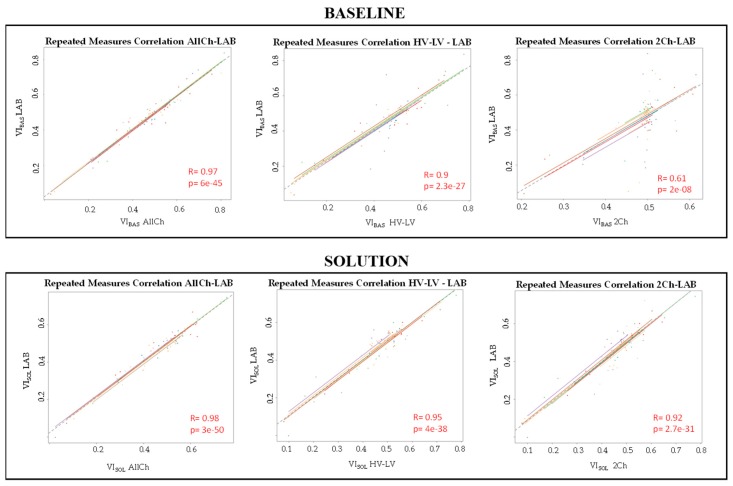
The figure reports the results of repeated measures correlation (rmcorr) analyses between the vigilance index of the Laboratory (LAB) configuration and the VI of the All-Channels (AllCh), High Vigilance-Low Vigilance (HV-LV) and 2-Channels (2Ch) Configuration, by using BASELINE and SOLUTION scenarios and the testing dataset. Each participant’s data and corresponding trend line are shown in different colours, while the dotted line indicates the common regression, and its sign of slope is the sign of R coefficient. All the correlations were positive (R > 0.61) and statistically significant (all *p* < 2 × 10^−8^).

Referring to the LAB configuration, the VI trends, computed on the *BASELINE* and *SOLUTION* (Figure 9), were both decreasing over time with a significant difference from the beginning to the end of the scenarios (*p* = 0.004 for the *BASELINE* and *p* = 8 × 10^−5^ for *SOLUTION* scenario). For each validation, the rmcorr analysis revealed that the VI of the LAB configuration had a significant (*p* < 2 × 10^−8^) and positive correlation (R > 0.61) with the VIs of the other configurations, as shown in Figure 10. 

## 4. Discussion

### 4.1. Summary of the Rationale of the Study 

Vigilance degradation could lead to very risky and hazardous conditions, especially in operative environments. For example, with the rapid development of civil aviation transportation and continuous increases in air traffic, we are reaching the capacity limits of the current air traffic management (ATM) systems. We have always relied on the human component to ensure air safety: air traffic controllers (ATCOs) ensure safe and efficient flow of air traffic in a portion of airspace, so-called sector. Thus, the capabilities of current ATM systems are constrained by the number of aircraft and the complexity of the situation experienced by ATCOs. To adequately consider the relevant additional complexity factors, researchers and experts have recognised the need to develop a better method to measure and predict complexity by combining several factors, including the airspace geometry, the characteristics of air traffic flow, and further operational constraints (e.g., environmental complexity) [76,77]. However, there is also a need for developing measures of complexity that can be used for addressing not only the characteristics of air traffic but also the cognitive aspects of the ATCO, such as vigilance. Cognitive aspects are important because future ATM systems will likely rely upon a high level of automation where human and machine coupling will be very tight. In fact, the introduction of automations moves the ATCOs from an active to a more passive role as automations will be able to provide separation and intervene in flight profiles without the controller’s intervention nor their permission. This future system architecture and automations level are seen as the way to envisage the future growth of air traffic while maintaining ATM safety. However, it is well known that prolonged supervisory activity impacts the controller’s sustained attention, or vigilance, levels by significantly reducing the reaction time and increasing the probability of missing important information or identify risky events [7,8,42,78].

The presented work aimed at developing and validating a low-invasive EEG-based vigilance index (VI) able to assess and track the user’s ongoing vigilance level. 

### 4.2. Considerations on Results 

In this regard, we first designed a controlled protocol employing a standard and scientifically recognised task to identify the EEG features strictly linked to vigilance degradation, and finally defined the EEG-based vigilance models. In particular, Experiment 1 was conducted by employing the 10-minute psychomotor vigilance task (PVT) [22,50,79] to identify the most significant brain features able to characterise and assess vigilance changes. The analyses of the reaction times (RTs) (Figure 3) revealed an increase with time on task and significant differences (*p* = 0.01 for PVT_1_ and *p* = 0.0017 for PVT_2_) between the first (${\mathrm{PVT}}_{1}^{1}$, ${\mathrm{PVT}}_{2}^{1}$—high vigilance conditions) and the ninth minutes (${\mathrm{PVT}}_{1}^{9}$, ${\mathrm{PVT}}_{2}^{9}$—low vigilance conditions) of the PVTs. The result was confirmed by experimental evidences in literature, and in fact, as expected, a decreasing in vigilance was related to increasing of RTs with time on task [9,18,21,76,80,81,82]. The comparison of the EEG PSDs between such *High* and *Low Vigilance* conditions of PVT_1_ highlighted that the parietal alpha, frontal beta and frontal gamma EEG rhythms could be the most significant features to assess vigilance changes over time. The alpha EEG rhythm is generally correlated to the attentional level, and it has been demonstrated that the higher the attention is, the higher desynchronization of the alpha activity is [83]. In the comparison between the beginning (${\mathrm{PVT}}_{1}^{1}$ and ${\mathrm{PVT}}_{2}^{1}$, respectively) and the end (${\mathrm{PVT}}_{1}^{9}$ and ${\mathrm{PVT}}_{2}^{9}$, respectively) of the tasks, it was found that PSD increased in the alpha band in response to a decrement of vigilance, therefore, the result was consistent with the scientific literature. Beta rhythm of the frontal gyrus is generally related to alertness and cognitive process in attention [84,85], and our results reported significant increases in the frontal PSD in the beta band between high and low vigilance conditions of PVT_1_. Gamma rhythm is normally involved in higher processing tasks and information processing, and the power in the gamma frequency band increases when individuals are mentally challenged [86]. The PSD increased significantly between high and low vigilance conditions of PVT_1_ when vigilance decreased. Experiment 1 also allowed the investigation of the possibility of defining the EEG channels’ configuration with the lowest number of electrodes (i.e. minima configuration) able to detect vigilance changes without significant performance reduction in terms of vigilance discrimination and classification with respect to configurations employing a higher number of electrodes. The first step was to consider all the available EEG channels except the ones related to motor activity (e.g., hand movements) and avoiding vigilance levels (e.g., high vs. low) discrimination due to the different number of movements during the execution of the PVTs. Then, to reduce the number of EEG channels containing eventual redundant information, we identified the most informative ones derived from the statistical comparison between the high and low vigilance conditions. At this point, the SWLDA was used to identify the most relevant spectral features able to discriminate the participants’ vigilance levels. This method allowed the realisation of configurations with different numbers of channels and aimed at defining the minima configuration (Figure 4). The results showed that the overall VI trend, computed for all the configurations, was a monotonous decreasing with significant differences between the beginning and the end of the PVTs. The results also confirmed that all the configurations were able to discriminate and to classify high and low levels of vigilance. In fact, the average classification (maxACC, i.e., maximum accuracy) and discrimination (AUC, i.e., area under curve) accuracy ranged from 75% to 89%, respectively (Figure 5). Additionally, the statistical analyses revealed that there were no differences of AUC and maxACC distributions among the different configurations (all *p* > 0.05). This means that it would be possible to use models with a low number of channels (i.e., two electrodes) without significant losses in the accuracy of vigilance discrimination and classification. Additionally, the LAB configuration (Figure 4), reported a non-significant reduction of 1.5% in terms of AUC and 3% in terms of maxACC with respect to the AllCh configuration. For such a reason and due to the low number of EEG sensors, it was chosen to validate the results in a realistic environment (Experiment 2). In particular, the LAB configuration in Figure 7 was obtained by using the common channels between the LAB configuration of Experiment 1 and the EEG montage used in Experiment 2. The results showed that the most appropriate EEG features to discriminate vigilance were the same as the ones derived from the laboratory study, that is parietal alpha, frontal beta and frontal gamma EEG rhythms (Figure 7). The SWLDA algorithm was then used on the two testing scenarios (i.e., *BASELINE* and *SOLUTION)* to gradually reduce the domain features and define the minima configuration. This method allowed the obtaining of the configurations of Figure 7, and their performance were estimated together with the LAB configuration (Figure 8). The VI computed for all the configurations showed a monotonous decreasing over time and significant differences (*p* < 0.05) from the beginning to the end of the ATM tasks, as well as in the Experiment 1. The rmcorr [75] also confirmed the presence of significant (all *p* < 2 × 10^−8^) positive correlation (R > 0) among the VI of LAB and others configurations (Figure 10) indicating that levels of vigilance were significantly discriminated and classified in the same direction. In addition, no significant differences (*p* > 0.05) between the configurations were found in terms of discrimination (AUC) and classification (maxACC) accuracy of vigilance. The average AUC and maxACC values estimated by using the LAB configuration were higher, or comparable to those of the other configurations realised with the features extracted from the ecological setting. This aspect is of particular importance because it demonstrates (i) how the EEG configuration derived from the laboratory dataset was capable of assessing vigilance changes in realistic settings involving professional personnel without significant accuracy reduction (i.e., inter-task, and inter-subjects validity), and (ii) the possibility to significantly reducing the number of sensors necessary to achieve good classification accuracy in realistic environment (from 61 to 2 EEG channels). In conclusion, the use of a minima configuration, together with increasingly reliable EEG dry electrodes, could speed up the usage of minimally invasive BCIs in real environments [87,88,89,90] and major comfort in the operative setting. 

### 4.3. Recommendations for Future Experimental Studies

Some limitations of the study should also be discussed. In fact, the experimental group of Experiment 2 was not gender-balanced as in Experiment 1 and, in addition, both groups had a small number of participants. In addition, using the 2Ch configuration, the model may be less immune to confound factors where other mental states variations may occur (e.g., mental workload). In addition, since the experimental settings of Experiment 2 were very realistic, the risk of collecting general noise-related data can be high. To deal with this critical issue, we employed several actions:

- First, we employed gel-based EEG electrodes to ensure low-impedance values over the entire experimental protocol and limit noise recording due to external interferences.
- Second, we started our study under highly controlled settings by choosing a laboratory environment, and a standard and controlled task for the vigilance assessment widely accepted and used in the scientific literature (e.g., psychomotor vigilance task (PVT)). The results derived from this study were then employed to design and evaluate the experiment in real settings.
- Third, we employed advanced signal processing techniques, starting with a conservative method (i.e., correcting the data through ICA) and then in a robust way (i.e., removing the epochs that cannot be corrected). In this regard, the average number of epochs removed from the EEG dataset was 18.5% ± 7.8% (mean ± standard deviation).
- Finally, we analysed the averaged PSDs values over a prolonged condition (1 min for the first experiment, 5 min for the second experiment) to mitigate the effects due to spurious outliers caused by casual events.

So, future studies could be useful to assess how vigilance indexes would vary accordingly with other mental states and find out the most appropriate model able to take into account this aspect. However, we may conclude that the results presented in our work are interesting and promising to be extended on enlarged and other professional personnel. The results obtained should be considered with caution because of the high risk of noise contamination, but these preliminary results seem interesting and encourage further research in this direction. Moreover, a possible development of the present work could concern the optimisation of the configuration of the sensors, eventually employing other neurophysiological signals and limiting the losses in discrimination and classification accuracy.

## 5. Conclusions 

The experiments conducted respectively in the laboratory and realistic environment allowed the identification of the most significant EEG features related to vigilance changes, in particular, the parietal alpha, frontal theta and frontal gamma rhythms. In both studies, starting from all the available EEG channels, different configurations were estimated to finally define the minima configuration, which is the lowest number of EEG channels per frequency band able to assess the user’s vigilance level without significant classification accuracy reduction. The results showed that the LAB configuration, derived from the laboratory dataset and participants, could be successfully used to characterise and assess vigilance changes even in different experimental settings (e.g., realistic air traffic control room) and on different participants (e.g., professional air traffic controllers) with no significant performance degradation. Finally, reducing the number of sensors from 61 to 2 EEG channels (e.g., minima configuration), we only found a slight reduction in classification accuracy. In particular, those losses ranged from 6% to 13% in the controlled setting, and from 4% to 17% in the ecological setting. The use of a low number of channels represents the advantages of both a lower amount of data to be processed (i.e., less computational demand), and a major comfort for the participants (i.e., fewer sensors, and less recording system invasiveness), especially during long tasks and operative environments. 

## Figures and Tables

**Table 1:** The table reports the number of those channels, selected by stepwise linear discriminant analysis (SWLDA) algorithm, able to detect vigilance changes in Experiment 1.

EEG Channels Configuration	Number of Channels
All-Channels (AllCh)	31
High Vigilance – Low Vigilance (HV-LV)	21
Laboratory (LAB)	19
2-Channels (2Ch)	2

**Table 2:** The table reports the number of those channels, selected by the stepwise linear discriminant analysis (SWLDA) algorithm, able to detect vigilance changes in Experiment 2.

EEG Channels Configuration	Number of Channels
All-Channels (AllCh)	13
High Vigilance – Low Vigilance (HV-LV)	11
Laboratory (LAB)	9
2-Channels (2Ch)	2
